# A Real-Time Signal-Based Wavelet Long Short-Term Memory Method for Length-of-Stay Prediction for the Intensive Care Unit: Development and Evaluation Study

**DOI:** 10.2196/71247

**Published:** 2025-08-20

**Authors:** Yiqun Jiang, Qing Li, Wenli Zhang

**Affiliations:** 1 Industrial and Manufacturing Systems Engineering, College of Engineering, Iowa State University, Ames, IA, United States; 2 Department of Information Systems and Business Analytics, Debbie and Jerry Ivy College of Business, Iowa State University, 3332 Gerdin Business Building, 2167 Union Drive, Ames, IA, 50011, United States, 1 5152942469

**Keywords:** ICU management, real-time vital signs, convolutional layer, signal processing, healthcare resource optimization, urgent care, intensive care unit

## Abstract

**Background:**

Efficient allocation of health care resources is essential for long-term hospital operation. Effective intensive care unit (ICU) management is essential for alleviating the financial strain on health care systems. Accurate prediction of length-of-stay in ICUs is vital for optimizing capacity planning and resource allocation, with the challenge of achieving early, real-time predictions.

**Objective:**

This study aimed to develop a predictive model, namely wavelet long short-term memory model (WT-LSTM), for ICU length-of-stay using only real-time vital sign data. The model is designed for urgent care settings where demographic and historical patient data or laboratory results may be unavailable; the model leverages real-time inputs to deliver early and accurate ICU length-of-stay predictions.

**Methods:**

The proposed model integrates discrete wavelet transformation and long short-term memory (LSTM) neural networks to filter noise from patients’ vital sign series and improve length-of-stay prediction accuracy. Model performance was evaluated using the electronic ICU database, focusing on 10 common ICU admission diagnoses in the database.

**Results:**

The results demonstrate that WT-LSTM consistently outperforms baseline models, including linear regression, LSTM, and bidirectional long short-term memory, in predicting ICU length-of-stay using vital sign data, achieving significant improvements in mean square error. Specifically, the wavelet transformation component of the model enhances the overall performance of WT-LSTM. Removing this component results in an average decrease of 3.3% in mean square error; such a phenomenon is particularly pronounced in specific patient cohorts. The model’s adaptability is highlighted through real-time predictions using only 3-hour, 6-hour, 12-hour, and 24-hour input data. Using only 3 hours of input data, the WT-LSTM model delivers competitive results across the 10 most common ICU admission diagnoses, often outperforming Acute Physiology and Chronic Health Evaluation IV, the leading ICU outcome prediction system currently implemented in clinical practice. WT-LSTM effectively captures patterns from vital signs recorded during the initial hours of a patient’s ICU stay, making it a promising tool for early prediction and resource optimization in the ICU.

**Conclusions:**

Our proposed WT-LSTM model, based on real-time vital sign data, offers a promising solution for ICU length-of-stay prediction. Its high accuracy and early prediction capabilities hold significant potential for enhancing clinical practice, optimizing resource allocation, and supporting critical clinical and administrative decisions in ICU management.

## Introduction

### Background and Significance

Efficient allocation of resources has emerged as a critical concern within the health care domain, with a specific focus on cost management. The effective administration of the intensive care unit (ICU) plays a pivotal role in attaining this objective [1]. ICUs have been reported to contribute significantly to a hospital’s financial allocation, ranging from 22% to 34% of the overall budget [2,3]. Hence, implementing improved management strategies for ICUs can effectively alleviate the financial burdens faced by the health care system.

Predicting patient outcomes in the ICUs has multifaceted implications, providing valuable supplementary information for medical professionals as they make critical clinical and administrative decisions (it is important to note that these predictions are intended to complement, not replace, the judgment of health care providers). First, the prediction of length of stay aids clinicians in strategizing ICU capacity planning [4]. Such predictions enable health care institutions to adeptly manage patient flow, thereby curtailing waiting durations for critically ill patients. This facilitates optimal bed turnover and efficient allocation of pivotal resources, including ventilators and staffing [5]. Second, the quantification and optimization of length-of-stay in critical care units are pivotal for enhancing patient outcomes and clinical quality [6]. An extended length of stay can potentially compromise the clinical quality within the ICU. Extended length of stay can exert undue pressure on ICU capacity, potentially resulting in the deferment of elective surgeries, which is both financially burdensome and detrimental to patient health [7]. Furthermore, it could escalate the urgency to refuse or postpone emergency admissions, potentially jeopardizing patient outcomes. Such scenarios could also inadvertently shift focus away from the gravely ill [7]. Accurate length-of-stay predictions empower intensivists to refine treatment strategies, enhancing patient outcomes while minimizing unwarranted interventions. Third, economic considerations are intricately linked with length-of-stay predictions. ICUs, by their inherent nature, are financially demanding, administering intricate interventions and mandating intensive clinician involvement for a niche patient cohort. An augmented length of stay inevitably monopolizes more ICU resources, thereby inflating costs. In a milieu where ICUs grapple with mounting pressures and financial resources are increasingly limited, the urgency to enhance the expediency and efficiency of critical care is paramount [8].

In an optimal setting, a patient outcome prediction model for intensive care would be deployed before any intervention [9]. However, in current clinical paradigms, the prediction is typically executed within the first 24 hours following ICU admission. This is primarily due to the necessity of integrating various patient-specific risk factors, including demographic information, diagnostic codes, and laboratory test results to accurately predict the outcome for individual patients [9]. The imperative to collate data from diverse sources poses challenges to the adaptability of existing methods for real-time predictions. The process is further complicated by the frequent occurrence of unidentified or “unknown” patients in the ICU, whose identities cannot be ascertained upon arrival at the hospital. As a result, demographic information, medical history, and related data remain undisclosed [10,11]. The absence of such vital information restricts the available input, compelling the model to rely solely on readily accessible data. In response to this challenge, researchers and practitioners advocate for the development of models that rely solely on real-time vital sign data, enabling predictive capabilities at any point during a patient’s stay in the ICU.

### Objectives

To address the critical need for efficient ICU length-of-stay prediction with limited patient information that can be updated in real time, this research aims to develop a predictive model for ICU length-of-stay based exclusively on real-time vital sign data. By leveraging real-time vital sign data, the study enables early and accurate length-of-stay predictions, facilitating improved ICU capacity planning, resource optimization, and enhanced patient care outcomes.

### Related Work

#### ICU Length-of-Stay Prediction

The importance of predicting the length of stay in the ICU has long been acknowledged, with numerous studies addressing this topic (Table 1). Predominantly, extant research tends to reduce the complexity of length-of-stay prediction into a binary classification problem, categorizing patients’ stays as either prolonged or nonprolonged. Nevertheless, such binary classifications lack the granularity necessary for medical practitioners to devise comprehensive care plans. Furthermore, while some regression models have been developed to predict the actual length of stay for patients in the ICU, these models are typically limited to predictive horizons of only the first 24 or 48 hours following ICU admission [12-16].

**Table 1.** Literature review.

Previous research	Data source	Data collection period	Type of prediction	Methods	Data type or feature used			
					Domain knowledge	Demographic and pre-ICU^a^ condition	Vital signs	Laboratory results
Mobley et al [17]	Records of patients discharged from a postcoronary care unit in early 1993.	24 h	Classification: length of stay (1 to 20 days).	NN^b^	✓	✓	✓	—^c^
Zimmerman et al [16]	In hospital	24 h	Regression	Linear regression	✓	✓	✓	✓
Van Houdenhoven et al [15]	In hospital	72 h	Regression	Linear regression	✓	✓	✓	✓
De Cocker et al [18]	In hospital	In hospital	Classification (if>2, if>5, if>7)	Risk model or hazard model	✓	✓	—	✓
Purushotham et al [13]	MIMIC-III^d^	24 h, 48 h	Regression	Deep learning models (MMDL^e^, FFN^f^, and RNN^g^)	✓	✓	✓	✓
Rajkomar et al [19]	In hospital	24 h, 48 h	Classification (if≥7 days)	LSTM^h^	✓	✓	✓	✓
Harutyunyan et al [20]	MIMIC-III	Real time, each hour after admission	Classification problem with 10 classes or buckets	LSTM	✓	✓	✓	✓
Khadanga et al [21]	MIMIC-II	48 h	Multiclass classification	CNN^i^ + LSTM	✓	—	—	—
Zebin and Chaussalet [22]	MIMIC-III	24 h	Binary classification	DNN^j^	✓	✓	—	—
Sotoodeh and Ho [23]	MIMIC-III	48 h	Regression	Hidden Markov model-based framework	—	✓	✓	✓
Ma et al [12]	In hospital	72 h	Regression	Decision tree	—	✓	✓	✓
Sheikhalishahi et al [14]	eICU^k^	24 h, 48 h	Regression	BiLSTM^l^	✓	✓	✓	✓
Alabbad et al [24]	In hospital	—	Classification	Random forest, gradient boosting, extreme gradient boosting, ensemble classifier	✓	✓	—	✓
Liu et al [25]	In hospital	—	Classification	Meta learning	✓	✓	✓	✓

a ICU: intensive care unit.

b NN: neural network.

c Not available.

d MIMIC-III: Medical Information Mart for Intensive Care III.

e MMDL: multimodal deep learning model.

f FFN: feedforward neural network.

g RNN: recurrent neural network.

h LSTM: long short-term memory.

i CNN: convolutional neural network.

j DNN: deep neural network.

k eICU: electronic intensive care unit.

l BiLSTM: bidirectional long short-term memory.

Given the critical condition of ICU patients, accurately assessing their health status—such as predicting their length of stay—is crucial, especially at time points specified by physicians rather than being limited to standardized intervals like 24 or 48 hours.

One of the principal challenges that existing models face is their reliance on multiple data sources, including domain-specific knowledge, demographic and pre-ICU condition data, vital signs, and laboratory results. Laboratory results, in particular, are often subject to processing delays, while domain knowledge relies on the interpretation of medical professionals, which can lack flexibility. In addition, demographic data may sometimes be unavailable, particularly in cases where patients’ identities are unknown. In contrast, bedside-monitored vital signs represent a readily available, real-time data source. However, current research uses vital sign series in a relatively superficial manner, typically using basic statistics such as mean, maximum, and minimum values [16], or categorizing them [26]. Nonetheless, vital sign time series data often exhibit highly complex patterns, which can vary significantly across different patient cohorts. Simplifying these series using basic statistics for categorical data severely limits downstream models’ ability to uncover valuable patterns and effectively leverage them for predicting ICU patients’ length of stay.

To address these limitations, our research endeavors to design a model that exclusively harnesses the power of 3 vital sign series to make real-time predictions, thereby offering a more granular and timely approach to ICU length-of-stay prediction with both decent accuracy and good generalizability.

#### Models Dealing With Time Series

The use of time series data for prediction has been a central focus in various domains, leading to the exploration and development of diverse models [27]. Traditional statistical models, particularly regression techniques, have historically played a crucial role in time series prediction [28]. These models leverage historical data patterns to make forecasts, providing a foundational framework for subsequent advancements in predictive modeling. However, their performance is constrained by their underlying hypothesis, as most datasets struggle to fulfill these assumptions.

The emergence of neural networks has revolutionized time series prediction. Recurrent neural networks are specifically designed to capture sequential dependencies within data [29]. Recurrent neural networks excel in modeling temporal relationships, making them well-suited for time series forecasting tasks [30]. However, they may face challenges when dealing with long-term dependencies due to the vanishing gradient problem [31]. The introduction of the long short-term memory (LSTM) model addresses this issue by incorporating memory cells that can retain information over extended sequences [32]. LSTMs have shown remarkable success in capturing complex temporal dependencies, making them popular for time series prediction tasks. However, they are not immune to challenges, particularly when the data is affected by noise [33]. The transformer architecture, originally designed for natural language processing tasks, has also found applications in time series prediction [34]. However, a notable challenge lies in the substantial amount of data often required for effective training [35]. In addition, for task-specific small datasets, transformers have demonstrated less competitiveness compared with LSTMs, as evidenced by research in the medical field [36]. Therefore, it appears that LSTM remains the preferred choice, notwithstanding its susceptibility to noise in time series data.

To address the susceptibility of LSTMs to noise, we propose using wavelet transform techniques as a preprocessing step and introduce the Wavelet-LSTM model. This approach aims to denoise time series data before feeding it into LSTM models, with the overarching goal of enhancing the robustness of LSTM models and improving their performance in the presence of noisy signals.

#### Wavelet Transformation in ICU Mortality Prediction

The application of wavelet transformation remains limited in ICU outcome prediction. A previous study by Wang et al [37] demonstrated that features extracted via wavelet transformation can be among the most informative compared with those derived from other signal processing techniques. However, their approach applied wavelet features in a handcrafted and static manner, rather than integrating them into an end-to-end learning framework. In addition, much of the existing literature does not fully use the rich, high-frequency information embedded in continuous vital sign data. The proposal of a wavelet LSTM model (WT-LSTM) addresses this gap by incorporating wavelet-transformed vital signs directly into the model architecture, enabling both noise reduction and multi-resolution pattern extraction in a fully data-driven way. This represents a key contribution of our work, as it combines advanced signal processing with deep learning to enhance predictive performance while maintaining practical applicability for real-time clinical decision support.

## Methods

### Model Structure

**Figure 1.**
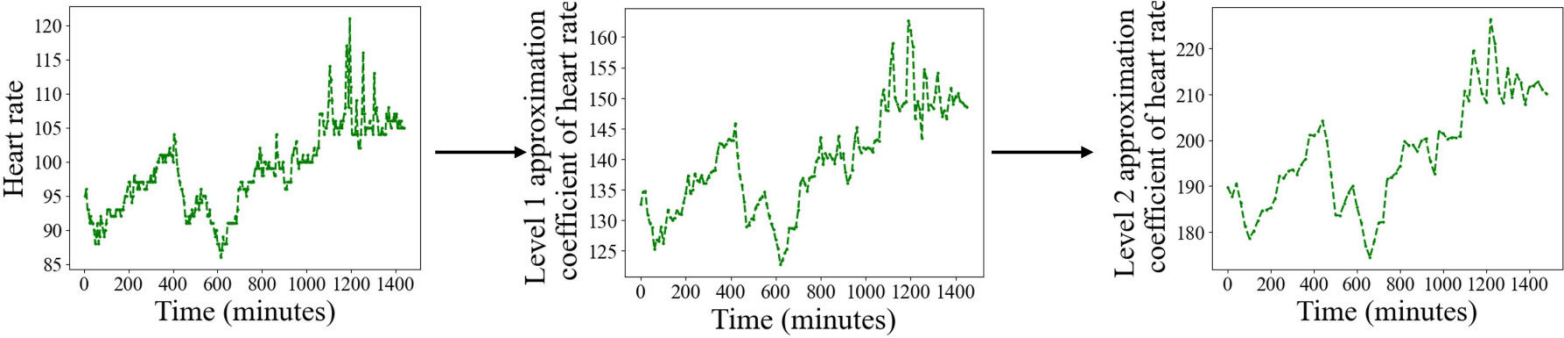
Alterations in vital sign series during level 2 discrete wavelet transform filter bank application.

The WT-LSTM model introduced in this study is primarily composed of 3 key components: a wavelet transform layer, an LSTM layer, and a linear fully connected layer. Using the vital sign series denoted by $V_{i}(t)\in 2^{N}$, where $i=1,...,m,t=1,...,n,N\in N^{+}$, we use a discrete wavelet transform (DWT) filter bank to discern the trends therein. The trends of the vital signs are encapsulated in the low-frequency component of the signal, while the high-frequency component is predominantly noise. Given a mother wavelet ψ(t), we can construct $g(t)=\frac{1}{\sqrt{2^{j}}}\psi (\frac{-t}{2^{j}})$ sampled at the points 1, $2^{j}$, $2^{2j}$,...,$2^{N}$, where j denotes the level of the DWT. In this investigation, we select j=2, thereby using a level 2 DWT filter bank. The coefficients of this filter bank align precisely with a wavelet coefficient of a discrete set of child wavelets for a given mother wavelet ψ(t). The $V_{i,t}$ series are channeled through a low-pass filter twice, culminating in the approximation coefficient $X_{i}(t)$ of the original signal (Figure 1).

By using DWT, high-frequency noise is filtered out, significantly enhancing the clarity of time series patterns in patients’ vital signs. These denoised sequences, $X_{i}(t)$ serve as the input for the LSTM network. At each time point, there are 3 input features: the values of heart rate, respiration, and oxygen saturation (SaO2), forming the input vector X at a specific moment, represented as $\left(V_{heartrate}V_{respiration}V_{sao2}\right)$.

**Figure 2.**
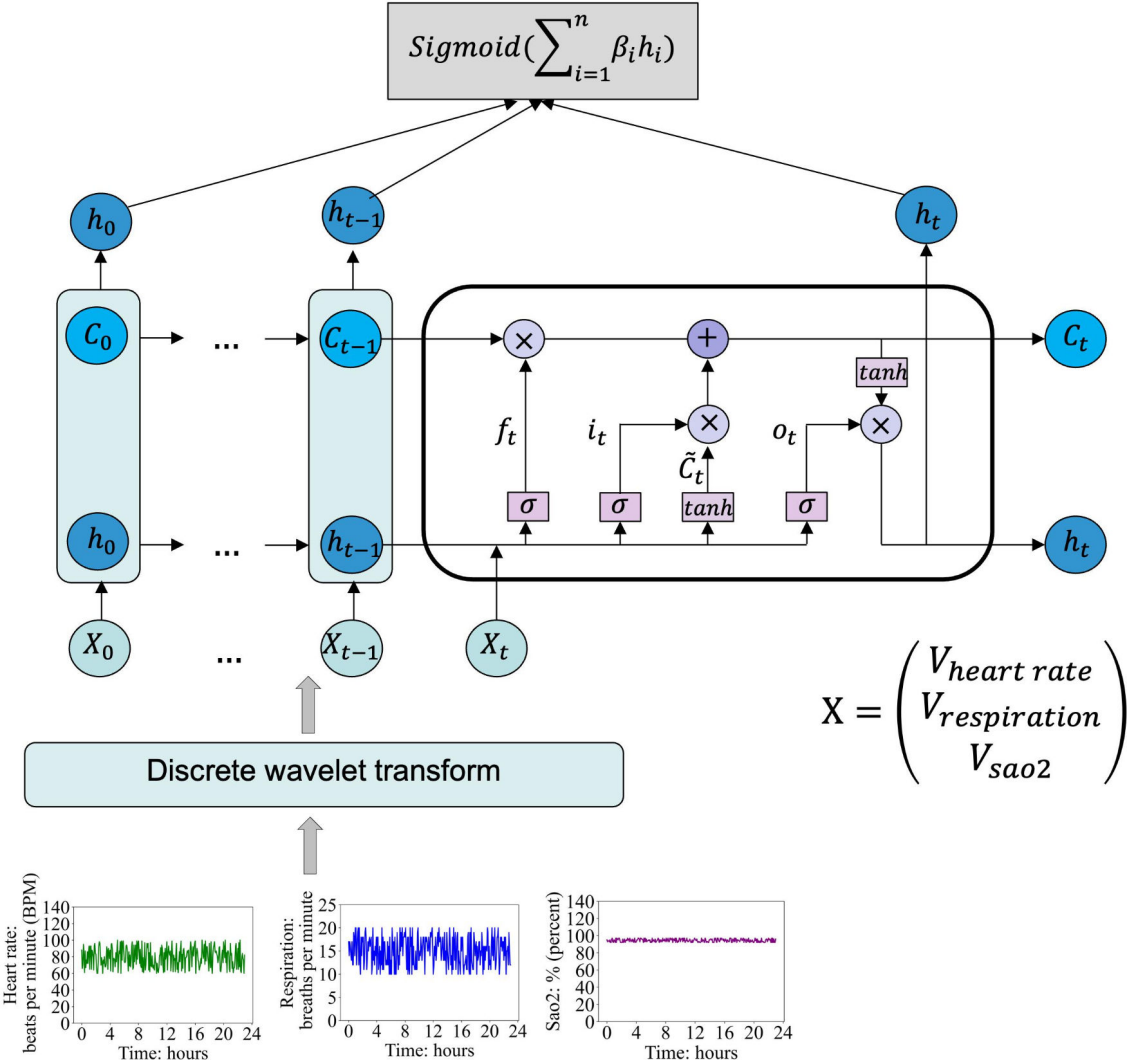
Wavelet long short-term memory model network structure.

The LSTM architecture efficiently uses temporal information from time series data by allowing past information to persist. For input $X_{i}$ at time stamp i, $\left(V_{heartrate,i}V_{respiration,i}V_{sao2,i}\right)$, an LSTM cell processes it as $f_{t}=\sigma (X_{t}*U_{f}+H_{t-1}*W_{f})$ , ${\tilde{C}}_{t}=tanh(X_{t}*U_{c}+H_{t-1}*W_{c})$, $i_{t}=\sigma (X_{t}*U_{i}+H_{t-1}*W_{i})$, $O_{t}=\sigma (X_{t}*U_{o}+H_{t-1}*W_{o})$, $C_{t}=f_{t}*C_{t-1}+i_{t}*{\tilde{C}}_{t}$, $H_{t}=O_{t}*{tanh(C}_{t})$, where W, U are the weight vectors for forget gate f, candidate c, i/p gate i and o/p gate O. $H_{t-1}$, $H_{t}$, $C_{t-1}$ and $C_{t}$ are the previous and current cell output and memories respectively (Figure 2). A linear layer is then applied to the LSTM cell outputs, akin to performing regression on these outputs. The final result of the model constitutes the predicted length-of-stay value.

The WT-LSTM model effectively combines the strengths of signal processing techniques, which excel at filtering noise from patients’ vital sign time series, with the robust pattern recognition capabilities of the LSTM model structure. Time series data of patients’ vital signs often contain significant noise due to varying disease pathologies, medical interventions, and device errors. The synergy of wavelet and LSTM components in the proposed model effectively mitigates the adverse effects of noise on LSTM performance, thereby enhancing its robustness and overall effectiveness.

### Data Description

The experiments were carried out using the electronic intensive care unit (eICU) database [38]. We extracted datasets encompassing patient records associated with the top 10 most prevalent diagnoses at ICU admission (Table 2). This selection allows for more reliable comparisons across clinically meaningful and commonly observed patient cohorts. The sole input to the model consisted of vital sign series, which constituted readily accessible real-time data from ICU bedside monitoring. These vital signs were typically interfaced as 1-minute averages and archived as 5-minute median values [37]. The vital signs used in this study presented themselves as periodic time-series data. Specifically, our analysis focused on 3 key vital signs: heart rate, respiration rate, and SaO2, recognized as the most pertinent indicators for ICU outcome prediction.

**Table 2.** Patient records with the top 10 most frequent diagnoses at intensive care unit admission.

Abbreviation	Diagnosis	Records, n
SP^a^	Sepsis, pulmonary	8862
MI^b^	Infarction, acute myocardial (MI)	7228
CVA^c^	CVA, cerebrovascular accident or stroke	6647
HF^d^	CHF^e^, congestive heart failure	6617
SR^f^	Sepsis, renal/UTI^g^ (including bladder)	5273
RD^h^	Rhythm disturbance (atrial, supraventricular)	4827
DK^i^	Diabetic ketoacidosis	4825
CA^j^	Cardiac arrest (with or without respiratory arrest; for respiratory arrest see Respiratory System)	4580
CABG^k^	CABG alone, coronary artery bypass grafting	4543
EB^l^	Emphysema or bronchitis	4494

a SP: sepsis, pulmonary.

b MI: myocardial infarction.

c CVA: cerebrovascular accident or stroke.

d HF: heart failure.

e CHF: congestive heart failure.

f SR: sepsis, renal.

g UTI: urinary tract infection.

h RD: rhythm disturbance.

i DK: diabetic ketoacidosis.

j CA: cardiac arrest.

k CABG: coronary artery bypass grafting.

l EB: emphysema or bronchitis.

For each experiment, the same patient cohort was used across all input time windows (eg, 3 h, 6 h, 12 h, and 24 h). To ensure temporal consistency and real-time applicability, only vital sign data recorded before the specified time point were used for model input. Patients with a length of stay shorter than the input window (eg, less than 24 h in the 24 h experiment) contributed their complete available data. For patients with longer stays, only the data from the specified time window were included. This approach preserved the real-world distribution of ICU lengths of stay while maintaining consistency in patient inclusion and ensuring that the model relied exclusively on data that would be available at the corresponding prediction time. The target variable, length of stay, was modeled and evaluated in units of days.

### Ethical Considerations

The eICU databases were deidentified, anonymized, and approved for sharing by the institutional review boards of both Beth Israel Deaconess Medical Center and the Massachusetts Institute of Technology. Data access was granted to an investigator after the completion of a National Institutes of Health course and successful passing of the associated human research participant protection examination. Given that the data are accessible to the public through the eICU database, the need for ethical approval and informed consent was waived. The contributing author, YJ, obtained the necessary authorization to access the anonymized dataset and oversaw the meticulous data extraction process.

### Training Details

The training, validation, and testing of our method follow the widely adopted hold-out validation procedure. Specifically, for each experiment, patient records were randomly split using stratified sampling based on the prediction target (mortality or length of stay). The data were partitioned into training (56.25%), validation (18.75%), and test (25%) sets. Each experiment was repeated 30 times with different random seeds to ensure robustness.

The specific training procedure and parameter settings of our method are summarized below. Our method was developed using PyTorch. We trained the WT-LSTM model for up to 100 epochs with early stopping based on validation loss. The optimizer used was Adam with learning rates tuned over 0.08, 0.1, 0.12, and 0.15. A batch size of 16 was used consistently across all runs. The best-performing model checkpoint (based on validation loss) was saved for evaluation on the test set. We have set torch.manual_seed(1) for reproducibility.

## Results

### Overview

In this section, we primarily present the outcomes of 3 key experiments. To commence, we juxtapose the results of the WT-LSTM model using mean square error (MSE), a commonly used metric for evaluating the performance of regression models, against benchmark methods used by existing research, including linear regression, LSTM, bidirectional long short-term memory (BiLSTM) using 24-hour vital sign data. Subsequently, we extend our analysis to experiments involving 3-hour, 6-hour, and 12-hour prediction intervals, thus showcasing the model’s real-time and early prediction capabilities. The results are compared with the best-performing ICU outcome prediction method currently used in ICUs, that is, the Acute Physiology and Chronic Health Evaluation (APACHE) IV system [16]. In addition, we present the length-of-stay prediction distributions for each patient cohort generated by our proposed model and draw comparisons with the predictions generated by the APACHE IV model.

### Comparison With Baselines

Previous studies on length-of-stay prediction have employed linear regression, LSTM, and BiLSTM models, which are adopted as baselines in our research, using vital sign series as inputs. This study conducts a comparative analysis between the performance of the WT-LSTM model, using 24-hour vital sign data, and the baselines to validate the effectiveness of our model (Table 3).

**Table 3.** Comparisons between wavelet long short-term memory model and benchmarks using 24 h vital signs as inputs.

Disease	Linear regression	BiLSTM^a^	LSTM^b^	WT-LSTM^c^	Improvement compared with LSTM, n (%)
HF^d^	17.61	14.75	13.84	13.24	0.6 (4.34)
CVA^e^	15.16	12.33	11.59	11.45	0.14 (1.21)
MI^f^	8.15	6.03	5.54	5.53	0.01 (0.18)
SP^g^	38.77	29.41	24.29	24.31	–0.02 (0.08)
SR^h^	15.24	9.99	9.08	8.84	0.24 (2.64)
RD^i^	10.69	7.71	6.66	6.02	0.64 (9.61)
DK^j^	4.08	2.38	2.39	2.37	0.02 (0.84)
CA^k^	38.77	22	20.04	19.22	0.82 (4.09)
CABG^l^	14.08	11.95	9.47	8.78	0.69 (7.29)
EB^m^	19.1	12.3	11.58	11.25	0.33 (2.85)

a BiLSTM: bidirectional long short-term memory.

b LSTM: long short-term memory.

c WT-LSTM: wavelet long short-term memory.

d HF: heart failure.

e CVA: cerebrovascular accident or stroke.

f MI: myocardial infarction.

g SP: sepsis, pulmonary.

h SR: sepsis, renal.

i RD: rhythm disturbance.

j DK: diabetic ketoacidosis.

k CA: cardiac arrest.

l CABG: coronary artery bypass grafting.

m EB: emphysema or bronchitis.

WT-LSTM outperforms all the baselines in 9 out of the 10 patient cohorts. In the remaining patient cohort (sepsis, pulmonary [SP]), while the WT-LSTM model did not achieve the best performance, its performance was very close to that of the best baseline in terms of MSE (24.29 for LSTM vs 24.31 for WT-LSTM) and outperformed the other 2 baselines. It is evident that when handling patients’ time series of vital sign data, WT-LSTM demonstrates its strengths compared with the baselines.

Furthermore, the performance disparity between the LSTM model and the WT-LSTM model serves as an evaluation of the denoising impact of wavelet transformation on vital sign series and its subsequent contribution to model performance. The inclusion of the wavelet transformation component in the WT-LSTM model results in an average improvement of 3.3% in prediction performance, measured through MSE. Notably, the most substantial enhancement is observed in the patient cohort with rhythm disturbance (RD), where performance improves by 9.61%.

These experimental results signify that, among all the models addressing vital sign data for the regression prediction task of ICU length-of-stay, WT-LSTM emerges as the superior choice. This aligns with our intuitive design of the model structure.

### Real-Time Prediction

The WT-LSTM model, by exclusively using vital sign series as its input, exhibits remarkable adaptability in facilitating real-time predictive capabilities. To illustrate this, a series of experiments is conducted to evaluate the model’s performance with varying lengths of patient time series data as input. In these experiments, we compare the WT-LSTM model with the widely used APACHE IV model, known for its credibility in predicting ICU outcomes, which relies on 24-hour data and includes demographic information, vital sign values, and laboratory results as inputs.

In comparisons using 24-hour data inputs across 10 distinct patient cohorts with various diagnoses, the WT-LSTM model outperforms the APACHE IV model in 9 out of the 10 cases (Table 4). Particularly noteworthy is the fact that, for 8 out of the 10 cohorts, the WT-LSTM model exhibits a significant performance enhancement, reducing the MSE by more than 10% compared with the APACHE IV model. In over half of the cohorts, the improvement surpasses the 20% mark.

**Table 4.** Real-time prediction comparison between wavelet long short-term memory and Acute Physiology and Chronic Health Evaluation IV.

Disease	APACHE^a^ IV (24 h)	3 h	WT-LSTM^b^ 3 h improvement compared with APACHE IV, n (%)	6 h	12 h	24 h	WT-LSTM 24 h improvement compared with APACHE IV, n (%)
HF^c^	12.80	15.23	−2.43 (−18.98)	15.20	15.05	13.24	−0.44 (3.44)
CVA^d^	11.58	12.72	−1.14 (−9.84)	12.71	12.67	11.45	0.13 (1.12)
MI^e^	6.85	6.10	0.75 (10.95)	6.06	6.03	5.53	1.32 (19.27)
SP^f^	34.67	29.92	4.75 (13.7)	29.76	29.69	24.31	10.36 (29.88)
SR^g^	11.14	10.36	0.78 (7)	10.32	10.26	8.84	2.3 (20.65)
RD^h^	7.54	7.96	−0.42 (−5.57)	7.91	7.72	6.02	1.52 (20.16)
DK^i^	2.72	2.44	0.28 (10.29)	2.44	2.39	2.37	0.35 (12.87)
CA^j^	30.43	24.77	5.66 (18.6)	24.37	23.48	19.22	11.21 (36.84)
CABG^k^	11.84	12.21	−0.37 (−3.13)	12.07	11.64	8.78	3.06 (25.84)
EB^l^	13.52	13.06	0.46 (3.4)	12.86	12.72	11.25	2.27 (16.79)

a APACHE: Acute Physiology and Chronic Health Evaluation.

b WT-LSTM: wavelet long short-term memory.

c HF: heart failure.

d CVA: cerebrovascular accident or stroke.

e MI: myocardial infarction.

f SP: sepsis, pulmonary.

g SR: sepsis, renal.

h RD: rhythm disturbance.

i DK: diabetic ketoacidosis.

j CA: cardiac arrest.

k CABG: coronary artery bypass grafting.

l EB: emphysema or bronchitis.

The results also demonstrate that the initial 3-hour vital sign data provides the most significant insights for the prediction of ICU length of stay. The early-phase vital sign patterns of patients carry substantial implications for the assessment of their clinical condition, and the extension of the input time series yields relatively marginal improvements in the model’s performance. Particularly, in the transition from 3-hour to 12-hour input intervals, the results demonstrate a noteworthy degree of similarity. However, when a 24-hour input interval is used, the model’s performance exhibits a more pronounced enhancement.

It is imperative to highlight that, for over half of the patient cohorts, the 3-hour results surpass those of the APACHE IV model. This observation underscores the model’s significant potential for early prediction.

### Prediction Distribution Comparison Between WT-LSTM and APACHE IV

To gain deeper insights into the distinctions in prediction results between WT-LSTM and APACHE IV, we generated plots that depict the predicted length-of-stay by both methods, in conjunction with the true values of the length-of-stay for the 10 distinct patient cohorts.

**Figure 3.**
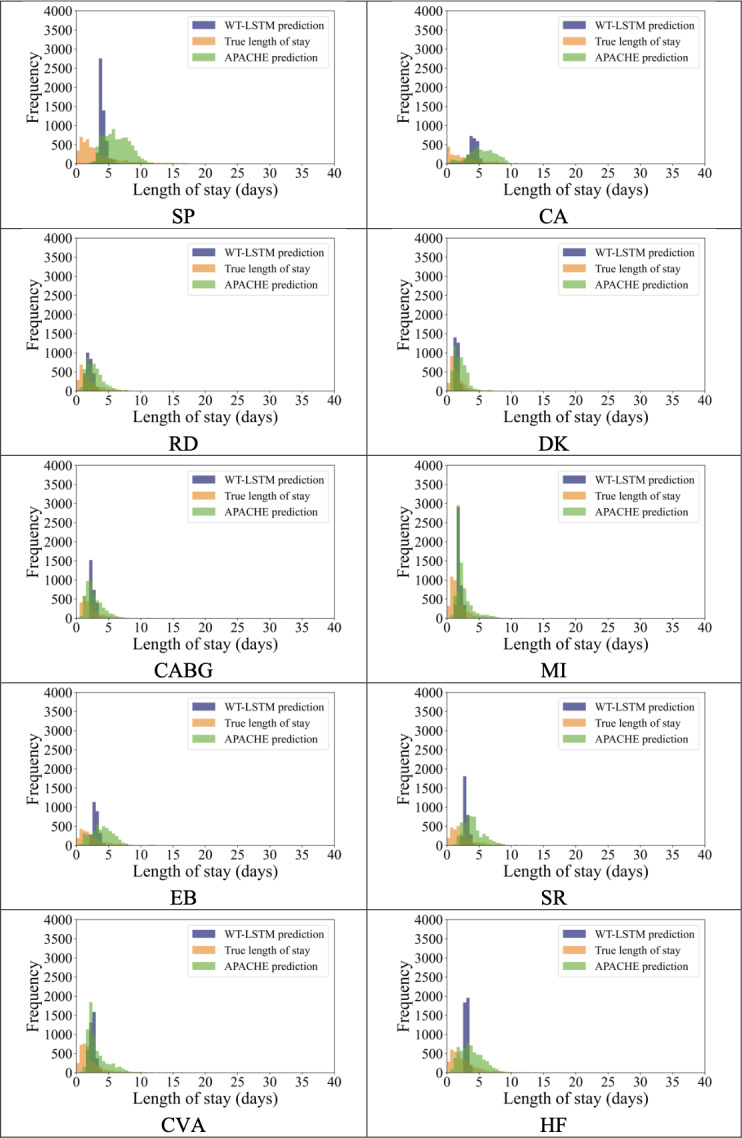
Predicted distribution of wavelet long short-term memory model with 3 h of vital signs versus Acute Physiology and Chronic Health Evaluation IV. CA: cardiac arrest; CABG: coronary artery bypass grafting; CVA: cerebrovascular accident or stroke; DK: diabetic ketoacidosis; EB: emphysema or bronchitis; HF: heart failure; MI: myocardial infarction; RD: rhythm disturbance; SR: sepsis, renal; SP: sepsis, pulmonary.

Observations gleaned underscore significant disparities in the patterns of length-of-stay predictions and actual values (Figure 3). Notably, the true values of length of stay exhibit a pronounced right-skewed distribution, whereas the predictions generated by APACHE IV tend to be more conservative in their estimates. WT-LSTM, on the other hand, positions itself between these 2 extremes, manifesting a propensity to predict values that gravitate toward the statistical average. The possible reason is that our method is based on deep learning, trained in a supervised manner using the true length of stay and optimized with a MSE loss function. Deep learning models optimized for MSE are often biased toward conservative predictions due to the bias-variance trade-off, which leads them to underpredict extreme values. For example, when a model trained with MSE makes an incorrect prediction on an extreme value, the squared error amplifies the loss significantly, discouraging the model from making such predictions.

**Figure 4.**
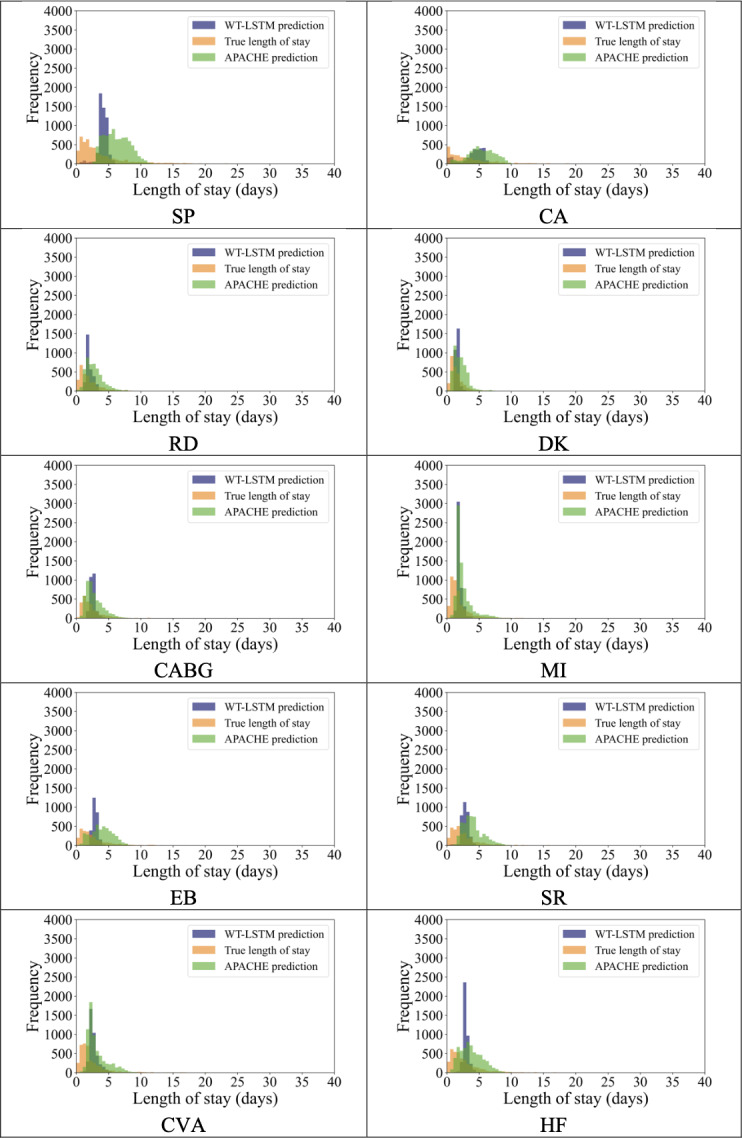
Predicted distribution of wavelet long short-term memory model with 24 hours of vital signs versus Acute Physiology and Chronic Health Evaluation IV. CA: cardiac arrest; CABG: coronary artery bypass grafting; CVA: cerebrovascular accident or stroke; DK: diabetic ketoacidosis; EB: emphysema or bronchitis; HF: heart failure; MI: myocardial infarction; RD: rhythm disturbance; SR: sepsis, renal; SP: sepsis, pulmonary.

We also compared the prediction distributions between the WT-LSTM model using the full 24-hour input series and APACHE IV (Figure 4). The results show that the distribution of predicted length-of-stay becomes more dispersed when 24 hours of data are used, with improved alignment to the true length-of-stay distribution. This suggests that increased input duration enhances the model’s sensitivity to patient-specific variation. However, the corresponding improvement in predictive accuracy, as measured by MSE, is relatively modest, as discussed previously—highlighting the strength of WT-LSTM’s early prediction capability, even when only short-term data are available.

These findings highlight that WT-LSTM, which relies solely on 3 hours of vital sign data, provides predictions that are highly competitive when compared with those generated by APACHE IV, which uses 24 hours of data. Furthermore, it has the potential to serve as an early warning system for monitoring the health conditions of patients.

## Discussion

### Limitations and Performance Interpretation

WT-LSTM has demonstrated its advantages in predicting ICU patients’ length of stay by using only real-time data that are readily accessible, achieving performance comparable with or better than most benchmark methods, including the best-performing method currently used in ICUs (ie, APACHE IV). However, it is essential to acknowledge its limitations. From the distribution comparisons presented in the results, it becomes evident that in certain patient cohorts, WT-LSTM tends to predict length-of-stay toward the mean, indicative of potential insufficient information.

One limitation of this study is the restriction to the top 10 ICU admission diagnoses in the eICU database. While this selection was made to ensure adequate sample sizes and manageable computational requirements, it may limit the generalizability of our findings to less common diagnoses or more heterogeneous ICU populations. Future work could extend the model to a broader patient population as resources permit.

The prediction results exhibit greater reliability and accuracy in patient cohorts with cardiac arrest (CA), RD, and diabetic ketoacidosis (DK), while showing relatively weaker predictions in cohorts with heart failure (HF), SP, and coronary artery bypass grafting (CABG). The primary reason for this discrepancy could be the diverse impacts that diseases have on the 3 vital signs. In disease cohorts where patient conditions significantly impact vital signs, distinguishing patients’ risk levels becomes challenging. For example, patients with HF often present with a rapid or irregular heartbeat, shortness of breath, and decreased SaO2 [39]. Similarly, patients with SP exhibit shortness of breath, an elevated heart rate, and decreased SaO2 [40]. Predicting outcomes using only these 3 vital signs proves challenging. In patient cohorts with diseases that have a limited impact on the 3 vital signs, such as CABG, which lacks clear signals from these vital signs, the performance of WT-LSTM is also limited. Conversely, for certain patient cohorts, the 3 adopted vital sign time series exhibit diverse patterns, and patient conditions have certain impacts on these vital signs; such variability can enhance the prediction capabilities of the WT-LSTM. For instance, patients with RD show varied patterns on respiration and SaO2 based on the type and severity of rhythm disturbance [41]. Similarly, DK and CA impact SaO2 differently based on the severity of acidosis and the involvement of respiratory arrest, respectively. This suggests that the predictive capability of WT-LSTM is influenced by the nature of the diseases and their respective impacts on vital signs. Recognizing these nuances is crucial for refining the model and improving its predictive performance across diverse patient cohorts. Furthermore, exploring additional vital signs specific to certain disease groups provides an opportunity to adapt the model to different conditions, potentially further improving its performance.

Besides, WT-LSTM’s exclusive reliance on vital sign data may lead to not fully capturing the complexity of certain clinical scenarios. For instance, critically ill patients undergoing prolonged interventions, such as mechanical ventilation, may exhibit relatively stable or normalized vital signs while still requiring extended ICU care. In such cases, the model may underestimate length of stay due to the absence of contextual clinical information. While the use of vital signs alone enhances the model’s applicability in real-time and data-limited settings, future work could explore the integration of additional variables such as medication use, intervention records, or clinical documentation to better account for factors not directly observable through vital sign patterns.

In addition, WT-LSTM’s exclusive reliance on vital sign data inherently limits its utility for individual-level prediction. The model performance at the individual scale is constrained by relatively low *R*² values (<10%) and wider prediction variance (higher root-mean-square error; Multimedia Appendix 1), as well as imperfect calibration. This is reflected in both the sharp distributional peaks seen in predicted length-of-stay. These patterns suggest that the model performs best when estimating average outcomes across a population but may struggle with edge cases or highly personalized clinical contexts. While this limitation does not preclude its use for operational or cohort-level applications, it is important to exercise caution in interpreting WT-LSTM predictions for individual patient decision-making. Future enhancements, such as incorporating auxiliary features or using distribution-aware loss functions, could help address this gap.

In Table 3, the BiLSTM model demonstrated worse performance than the unidirectional LSTM across most patient cohorts. This finding may be attributed to the temporal nature of ICU data, where the most predictive information is often concentrated in the initial hours following admission. Unlike LSTM, which processes data in a forward-looking manner aligned with real-time clinical decision-making, BiLSTM leverages both past and future time steps—an assumption that may not hold in real-world ICU settings where future observations are unavailable. In addition, BiLSTM’s bidirectional architecture increases model complexity and may lead to overfitting when training data is relatively limited, especially when early vital signs dominate the input. These factors suggest that BiLSTM’s backward temporal dependency may dilute the predictive strength of early signals, thereby reducing its overall effectiveness in this context. This further supports the design choice of WT-LSTM, which retains a unidirectional structure while enhancing temporal feature extraction through wavelet transformation.

Beyond individual-level predictions, the WT-LSTM model also has potential applications in ICU benchmarking and operational assessment. Early and accurate predictions of ICU length of stay can inform capacity planning, staffing allocation, and overall resource usage—key metrics in evaluating ICU efficiency. Previous works [42-45] have demonstrated the value of data-driven approaches in benchmarking ICU performance across institutions. By relying solely on real-time vital signs, our model offers a lightweight and scalable solution that could support these benchmarking efforts, particularly in settings with limited access to comprehensive electronic health record data. Integrating such predictive tools into ICU management workflows may help improve institutional comparisons, optimize throughput, and enhance system-level decision-making.

### Conclusions

This study introduces a novel model, WT-LSTM, which incorporates signal processing techniques to augment the performance of LSTM cells, specifically for the purpose of predicting ICU length-of-stay. WT-LSTM operates exclusively on readily available vital sign data, which effectively addresses 2 significant challenges in current research for ICU outcome prediction: real-time prediction capabilities and the lack of important information for unidentified patients.

The model’s performance is rigorously evaluated using the eICU database, focusing on patient records related to the top 10 most frequently diagnosed conditions. It is benchmarked against existing methods, including APACHE IV, which is a widely recognized and best-performing method currently used for ICU outcome prediction. Remarkably, when using 24-hour heart rate, respiration, and SaO2 time series as input, WT-LSTM significantly outperforms APACHE IV across most patient cohorts. Strikingly, even with just 3-hour vital sign series, WT-LSTM surpasses APACHE IV—despite the latter using 24 hours of data—in more than half of the patient cohorts.

The predictive distribution generated by WT-LSTM exhibits a tendency to predict values closer to the statistical average, offering a meaningful indicator for the early detection of changes in patients’ health conditions. This capacity to predict ICU length-of-stay both early and accurately not only provides valuable insights into patients’ health statuses, thereby benefiting health care providers in their clinical practice, but also offers guidance for optimizing the allocation of ICU resources. Ultimately, these advancements hold the potential to contribute significantly to healthcare management.

## Supplementary material

10.2196/71247 Multimedia Appendix 1 Additional tables and figures.

## Abbreviations

APACHE: Acute Physiology and Chronic Health Evaluation
BiLSTM: bidirectional long short-term memory
CA: cardiac arrest
CABG: coronary artery bypass grafting
DK: diabetic ketoacidosis
DWT: discrete wavelet transform
eICU: electronic intensive care unit
HF: heart failure
ICU: intensive care unit
LSTM: long short-term memory
MSE: mean square error
RD: rhythm disturbance
SaO2: oxygen saturation
SP: sepsis, pulmonary
WT-LSTM: wavelet long short-term memory model
