# Supplementary material for: A Real-Time Signal-Based Wavelet Long Short-Term Memory Method for Length-of-Stay Prediction for the Intensive Care Unit: Development and Evaluation Study
Source: JMIR AI. 2025 Aug 20;4:e71247. doi: 10.2196/71247 (PMC12367335; doi:10.2196/71247)
Supplement: Multimedia Appendix 1 [file ai-v4-e71247-s001.docx]

## Appendices

## S1. Benchmark Comparison Using Full Clinical Data

To further contextualize the performance of the proposed WT-LSTM model, we implemented a benchmark model trained on full clinical data collected within the first 24 hours of ICU admission. This benchmark leverages the same types of variables commonly used in traditional severity scoring systems, such as SAPS III [1], including demographic features (e.g., age, sex), vital signs, intervention status (e.g., ventilation, dialysis), and laboratory measurements.

Due to substantial variability in data completeness across clinical variables and patient cohorts, we applied Multiple Imputation by Chained Equations (MICE) to address missingness. Table S1 summarizes the missing data rates for each variable across the ten most common ICU admission diagnoses in the eICU database. As shown, while demographic and intervention variables were generally complete, several laboratory values—including pH, albumin, and arterial blood gas parameters—had high missing rates in multiple cohorts (e.g., >80% in CA and SP cohorts). These patterns illustrate the challenge of using full clinical data in real-time applications, where such values may not be immediately available.

A linear regression model was trained using the imputed dataset and evaluated by ten-fold cross validation for each cohort. Table S2 compares the Mean Squared Error (MSE) between the benchmark model and the WT-LSTM model. In a few cohorts, such as DK and CVA, the benchmark model achieved slightly lower MSE. However, in several other cohorts—including SP, CA, and RD—the WT-LSTM model performed significantly better, despite relying solely on readily available real-time vital sign data. These results underscore WT-LSTM's practical advantage in environments with limited data availability, and its ability to produce competitive predictions using a lightweight, clinically feasible input set.

Table S1.1. Missing Data Rates (%) by Variable and Patient Cohort

|  | SR | CVA | MI | SP | HF | RD | DK | CA | CABG | EB |
| --- | --- | --- | --- | --- | --- | --- | --- | --- | --- | --- |
| gender | 0.00% | 0.00% | 0.00% | 0.00% | 0.00% | 0.00% | 0.00% | 0.00% | 0.00% | 0.00% |
| age | 0.00% | 0.00% | 0.00% | 0.00% | 0.00% | 0.00% | 0.00% | 0.00% | 0.00% | 0.00% |
| intubated | 0.00% | 0.00% | 0.00% | 0.00% | 0.00% | 0.00% | 0.00% | 0.00% | 0.00% | 0.00% |
| vent | 0.00% | 0.00% | 0.00% | 0.00% | 0.00% | 0.00% | 0.00% | 0.00% | 0.00% | 0.00% |
| dialysis | 0.00% | 0.00% | 0.00% | 0.00% | 0.00% | 0.00% | 0.00% | 0.00% | 0.00% | 0.00% |
| eyes | 0.76% | 1.05% | 0.56% | 1.38% | 1.21% | 0.98% | 0.62% | 5.47% | 3.60% | 1.45% |
| motor | 0.76% | 1.05% | 0.56% | 1.38% | 1.21% | 0.98% | 0.62% | 5.47% | 3.60% | 1.45% |
| verbal | 0.76% | 1.05% | 0.56% | 1.38% | 1.21% | 0.98% | 0.62% | 5.47% | 3.60% | 1.45% |
| meds | 0.32% | 0.51% | 0.31% | 0.52% | 0.48% | 0.80% | 0.43% | 0.73% | 0.56% | 0.79% |
| urine | 55.68% | 47.15% | 40.40% | 58.30% | 49.97% | 48.55% | 37.24% | 57.24% | 59.37% | 52.67% |
| wbc | 18.52% | 30.29% | 25.63% | 19.60% | 28.26% | 34.53% | 35.88% | 19.69% | 8.20% | 28.61% |
| temperature | 4.84% | 4.96% | 5.61% | 5.46% | 6.94% | 8.10% | 7.25% | 10.40% | 6.64% | 6.43% |
| respiratory rate | 0.76% | 0.86% | 1.11% | 0.82% | 0.93% | 1.14% | 0.77% | 1.50% | 0.40% | 1.18% |
| sodium | 14.08% | 28.60% | 21.80% | 15.28% | 19.71% | 29.21% | 2.41% | 14.37% | 12.34% | 23.82% |
| heart rate | 0.32% | 0.37% | 0.51% | 0.59% | 0.48% | 0.66% | 0.34% | 0.84% | 0.25% | 0.72% |
| mean bp | 0.36% | 0.76% | 0.59% | 0.84% | 0.75% | 0.91% | 0.58% | 1.43% | 0.31% | 0.87% |
| ph | 82.34% | 89.87% | 92.41% | 64.52% | 71.71% | 93.43% | 88.85% | 40.76% | 29.03% | 57.76% |
| hematocrit | 17.46% | 29.69% | 24.12% | 18.49% | 27.48% | 33.52% | 35.15% | 18.12% | 4.22% | 28.06% |
| creatinine | 14.60% | 28.83% | 21.32% | 15.88% | 20.05% | 29.21% | 4.28% | 15.21% | 12.27% | 24.52% |
| albumin | 45.74% | 67.67% | 65.83% | 45.79% | 58.88% | 66.27% | 62.42% | 44.78% | 79.80% | 63.70% |
| pao2 | 82.34% | 89.87% | 92.41% | 64.52% | 71.71% | 93.43% | 88.85% | 40.76% | 29.03% | 57.76% |
| pco2 | 82.34% | 89.87% | 92.41% | 64.52% | 71.71% | 93.43% | 88.85% | 40.76% | 29.03% | 57.76% |
| bun | 14.56% | 29.34% | 21.92% | 16.07% | 20.11% | 29.53% | 4.20% | 15.19% | 15.35% | 24.61% |
| glucose | 7.47% | 15.48% | 14.29% | 8.51% | 10.14% | 20.16% | 0.98% | 9.83% | 0.69% | 13.03% |
| bilirubin | 51.53% | 70.14% | 68.94% | 50.14% | 64.11% | 69.42% | 69.69% | 47.48% | 82.28% | 67.12% |
| fio2 | 82.34% | 89.87% | 92.41% | 64.52% | 71.71% | 93.43% | 88.85% | 40.76% | 29.03% | 57.76% |

Table S1.2. MSE Comparison Between Full Clinical Data Model and WT-LSTM

| Disease | full clinical | WT-LSTM |
| --- | --- | --- |
| HF | 12.53 | 13.24 |
| CVA | 10.38 | 11.45 |
| MI | 5.99 | 5.53 |
| SP | 55.41 | 24.31 |
| SR | 9.00 | 8.84 |
| RD | 6.75 | 6.02 |
| DK | 2.18 | 2.37 |
| CA | 25.35 | 19.22 |
| CABG | 9.97 | 8.78 |
| EB | 12.02 | 11.25 |

## S2. Additional Discrimination Metrics and Calibration Plots

To provide a comprehensive evaluation of the model’s performance, we report additional performance metrics—Root Mean Squared Error (RMSE) and R²—alongside Mean Squared Error (MSE). These metrics are reported for each of the ten diagnostic cohorts using both 3-hour and 24-hour vital sign input windows.

As shown in Table S2.1, 24-hour inputs result in modest but consistent improvements across MSE, RMSE, and R², reflecting the benefit of longer monitoring duration. However, even with only 3-hour inputs, WT-LSTM achieves competitive results, reinforcing its utility for early prediction. These results offer a benchmark for future ICU LOS prediction models and underscore WT-LSTM's potential for real-time clinical decision support.

Table S2.1. Performance metrics

| Disease | 3H | | | 24H | | |
| --- | --- | --- | --- | --- | --- | --- |
|  | MSE | RMSE | R2 | MSE | RMSE | R2 |
| HF | 15.230 | 3.793 | 0.008 | 13.240 | 3.640 | 0.011 |
| CVA | 12.720 | 3.429 | 0.031 | 11.450 | 3.380 | 0.032 |
| MI | 6.100 | 2.540 | 0.026 | 5.530 | 2.350 | 0.028 |
| SP | 29.920 | 6.275 | 0.014 | 24.310 | 4.930 | 0.015 |
| SR | 10.360 | 3.243 | 0.030 | 8.840 | 2.970 | 0.032 |
| RD | 7.960 | 2.648 | 0.042 | 6.020 | 2.453 | 0.052 |
| DK | 2.440 | 1.639 | 0.036 | 2.370 | 1.540 | 0.040 |
| CA | 24.770 | 4.965 | 0.106 | 19.220 | 4.380 | 0.109 |
| CABG | 12.210 | 3.234 | 0.004 | 8.780 | 2.960 | 0.005 |
| EB | 13.060 | 3.547 | 0.009 | 11.250 | 3.350 | 0.009 |

In addition to these numerical metrics, we present calibration plots to visually assess the alignment between predicted and observed ICU LOS values. These plots, based on WT-LSTM predictions using 24-hour vital sign inputs, are shown in Figure S2.1. Calibration plots offer valuable insight into the agreement between model between predicted values $\hat{y}$ and actual observed outcomes $y$ across different segments of the outcome distribution. The calibration plot assesses whether the model systematically overestimates or underestimates the outcome across its range, providing a visual diagnostic of prediction bias.

To construct the calibration curve, the range of observed outcomes is partitioned into $K$ discrete bins using quantile binning, such that each bin contains approximately the same number of observations based on empirical percentiles. For each bin $k=1,...,K$, the average observed outcome and the corresponding average predicted value are computed. These bin-level means, $\underline{y_{k}}$​ and $\underline{\hat{y}_{k}}$​, respectively, are then plotted with the average observed outcome on the x-axis and the average predicted value on the y-axis. A 45-degree reference line (slope = 1, intercept = 0) is included to represent perfect calibration.

Deviations of the plotted points from this identity line indicate miscalibration; points above the line suggest the model underestimates the outcome in that range, while points below indicate overestimation. The degree to which the calibration curve aligns with the identity line reflects the model’s calibration performance across different outcome levels.

Overall, we observe that the predicted values generally align near the diagonal for mid-range LOS values, indicating good calibration around the population mean. However, for extreme cases—either very short or very long observed LOS—the predictions tend to regress toward the mean, showing less accuracy at the tails of the distribution. This pattern aligns with the findings presented in Figure 4 of the main text, which illustrates that WT-LSTM predictions exhibit a concentrated distribution around central LOS values. Such behavior is common in deep learning models optimized using MSE loss, which may inherently favor minimizing overall error by producing conservative estimates, especially when faced with highly skewed outcome distributions.

These calibration plots highlight the model’s strength in handling typical cases and suggest that future work may improve performance on outlier cases by incorporating distribution-aware training objectives or auxiliary clinical features that capture case complexity more explicitly.

| 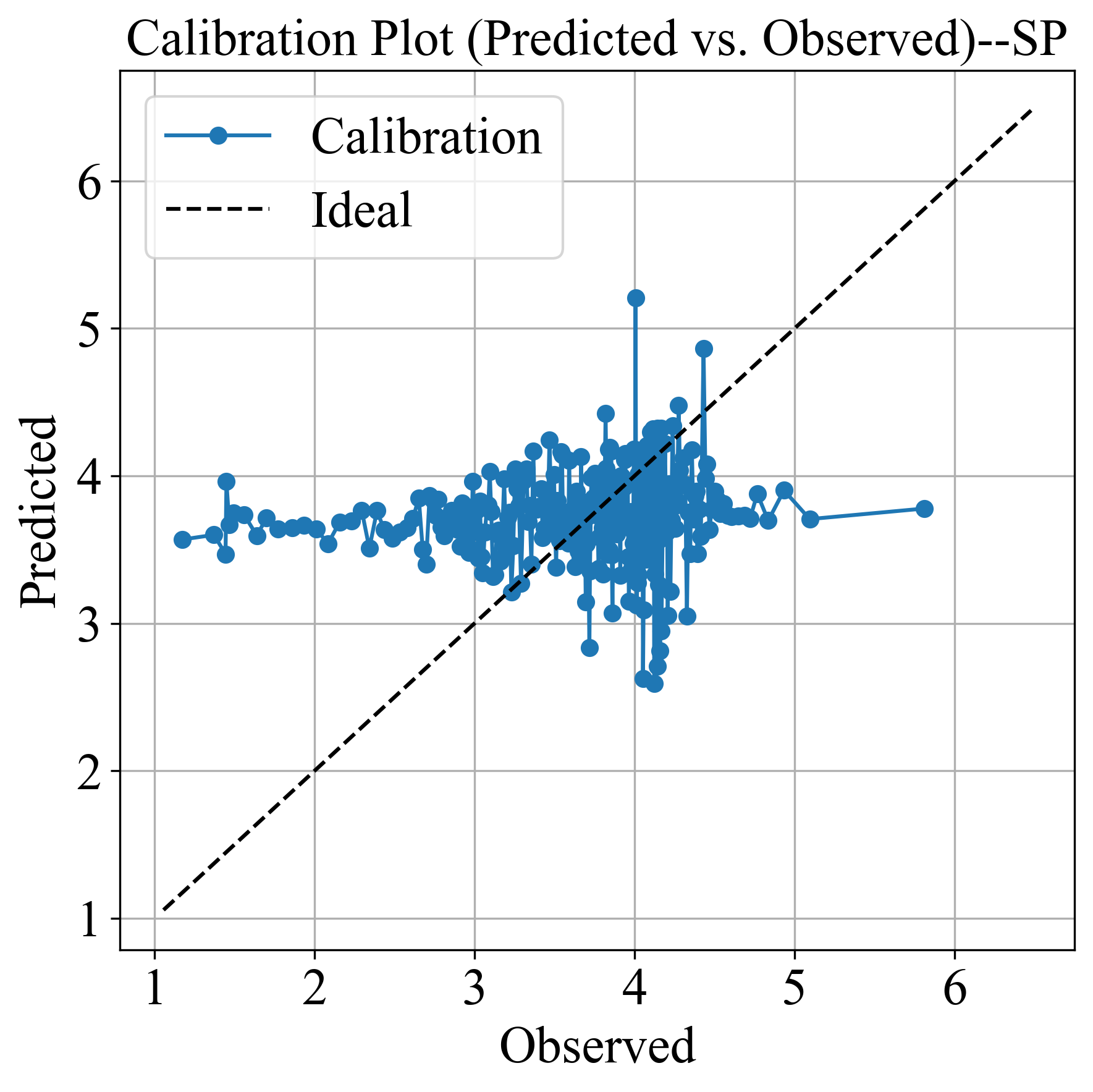SP | 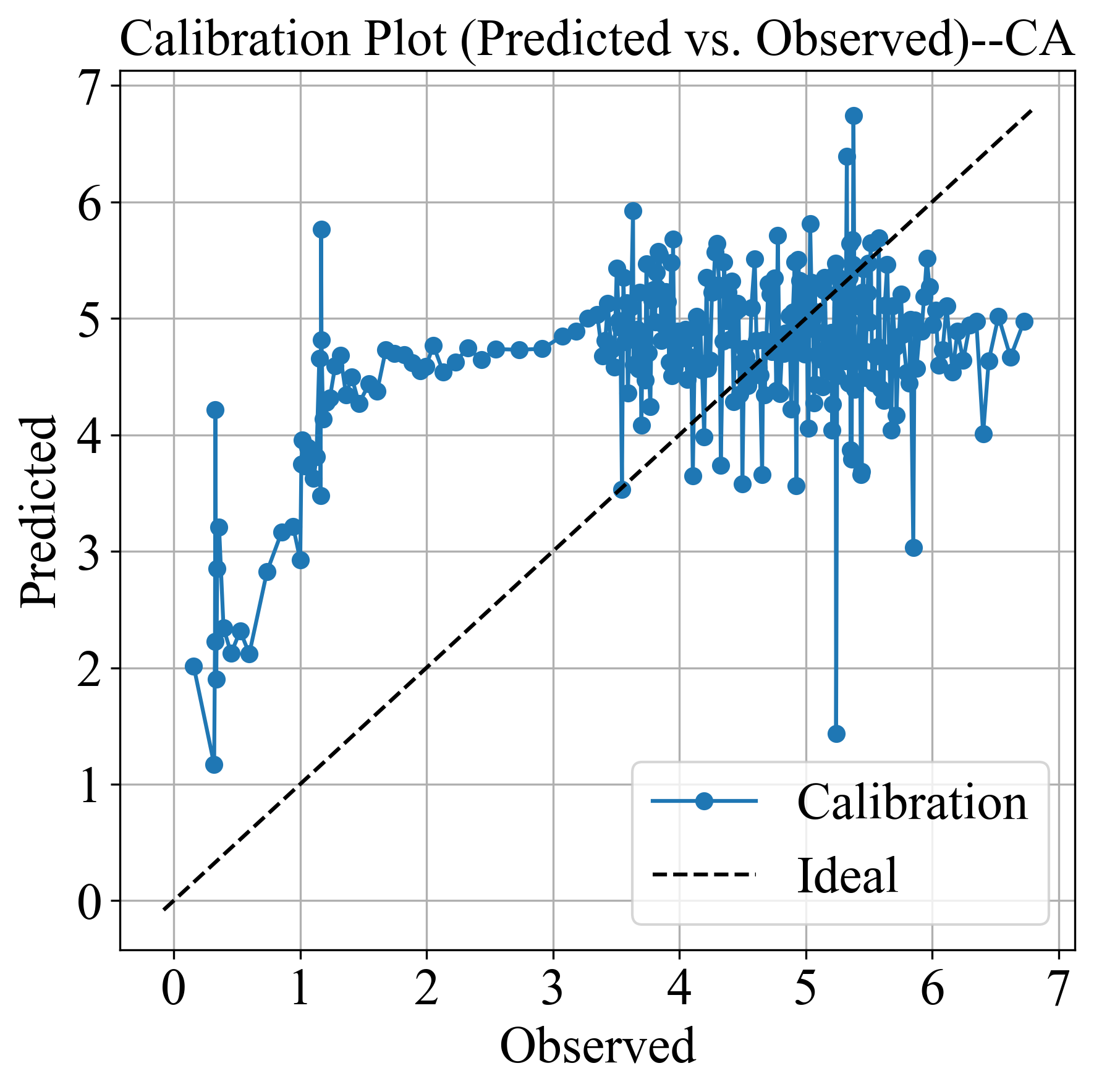CA |
| --- | --- |
| 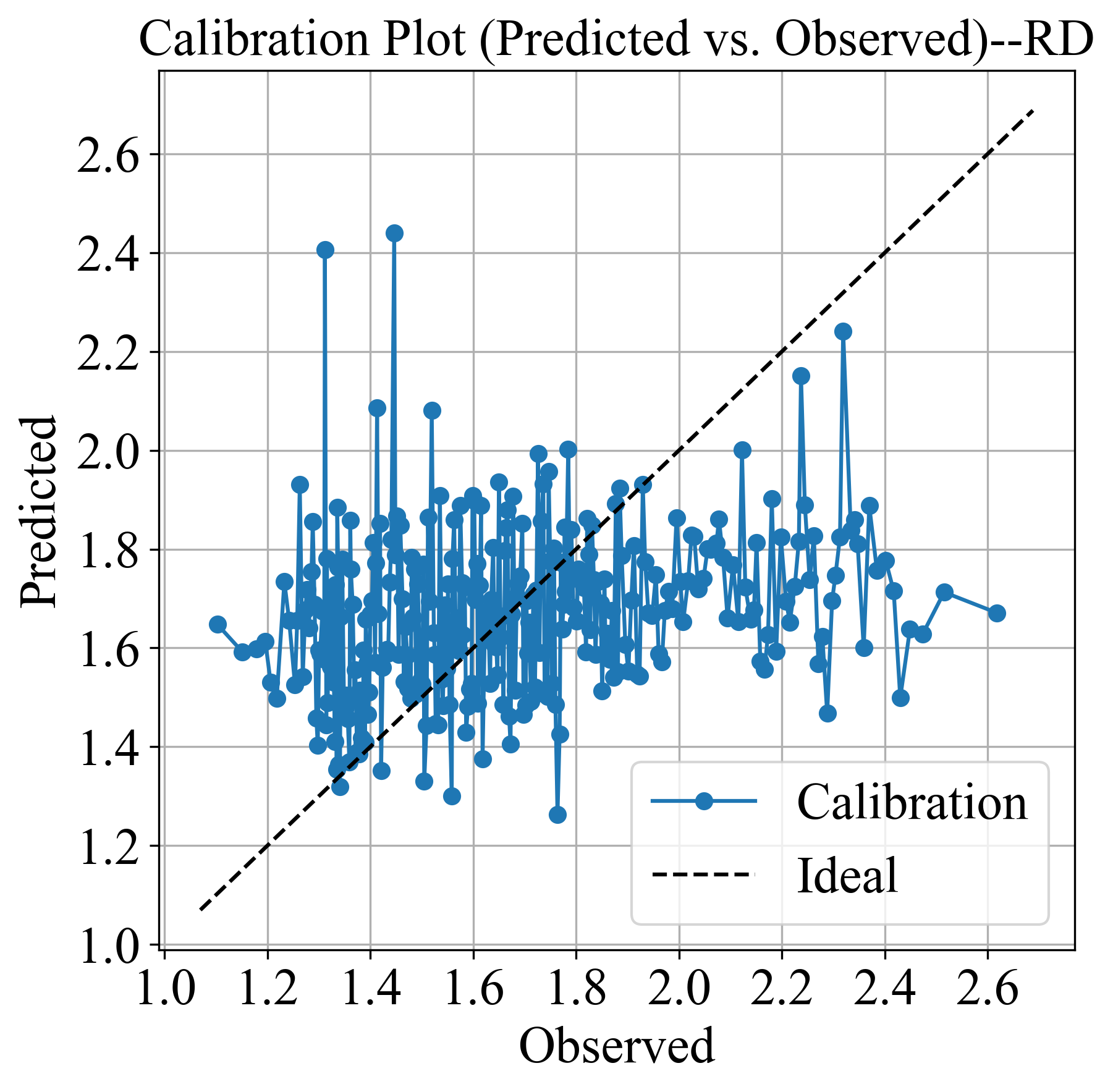RD | 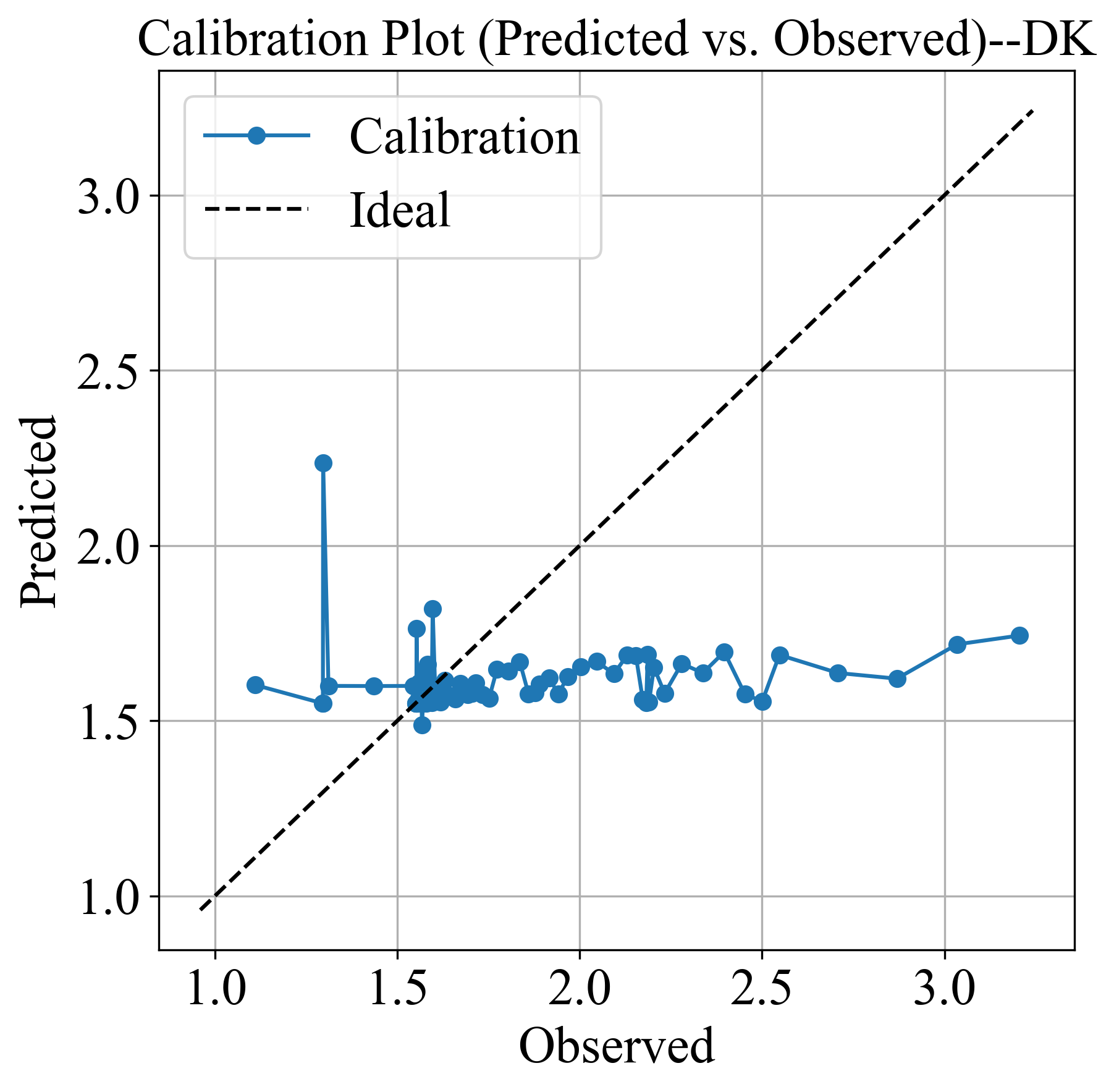DK |
| 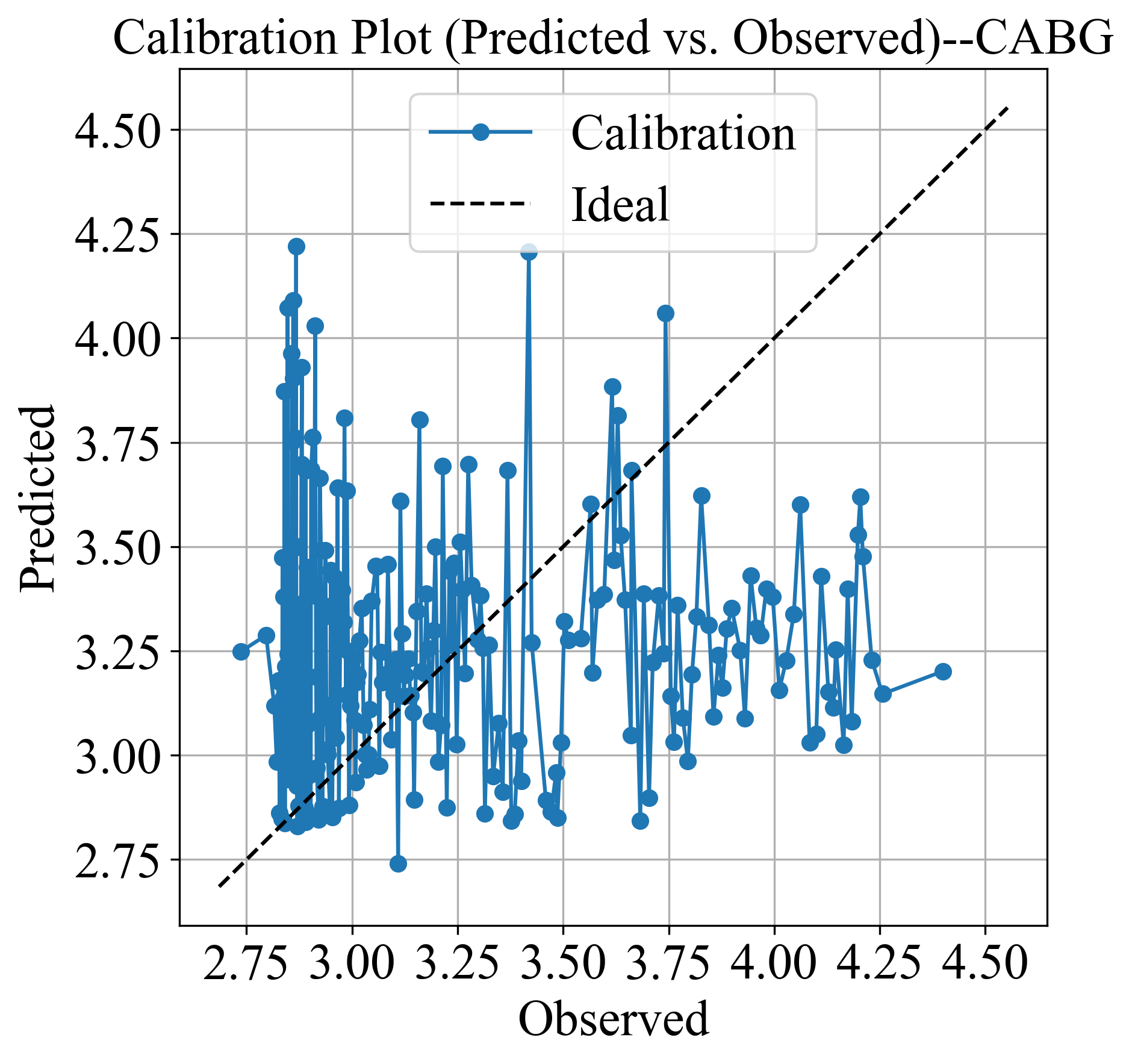CABG | 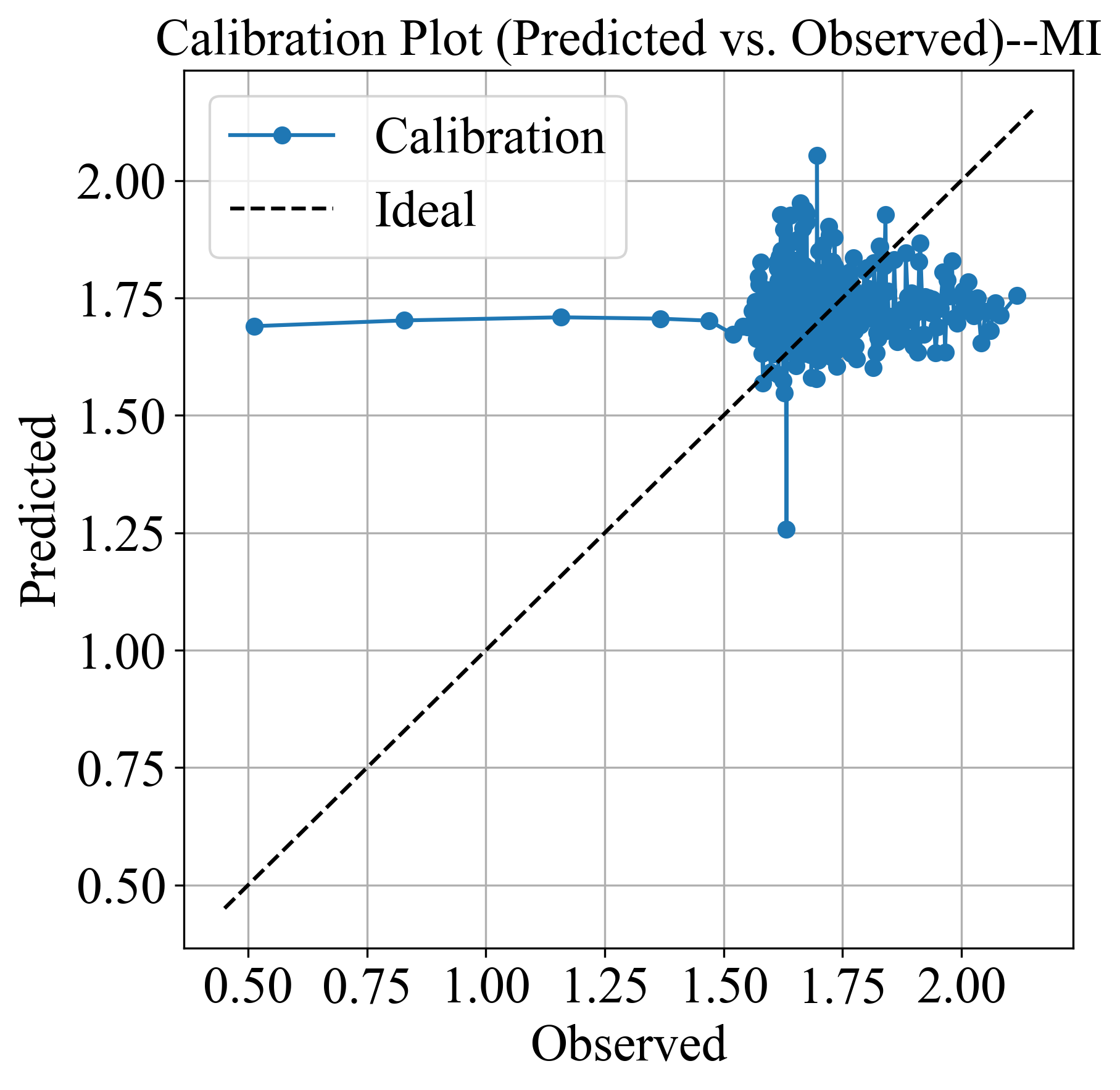MI |
| 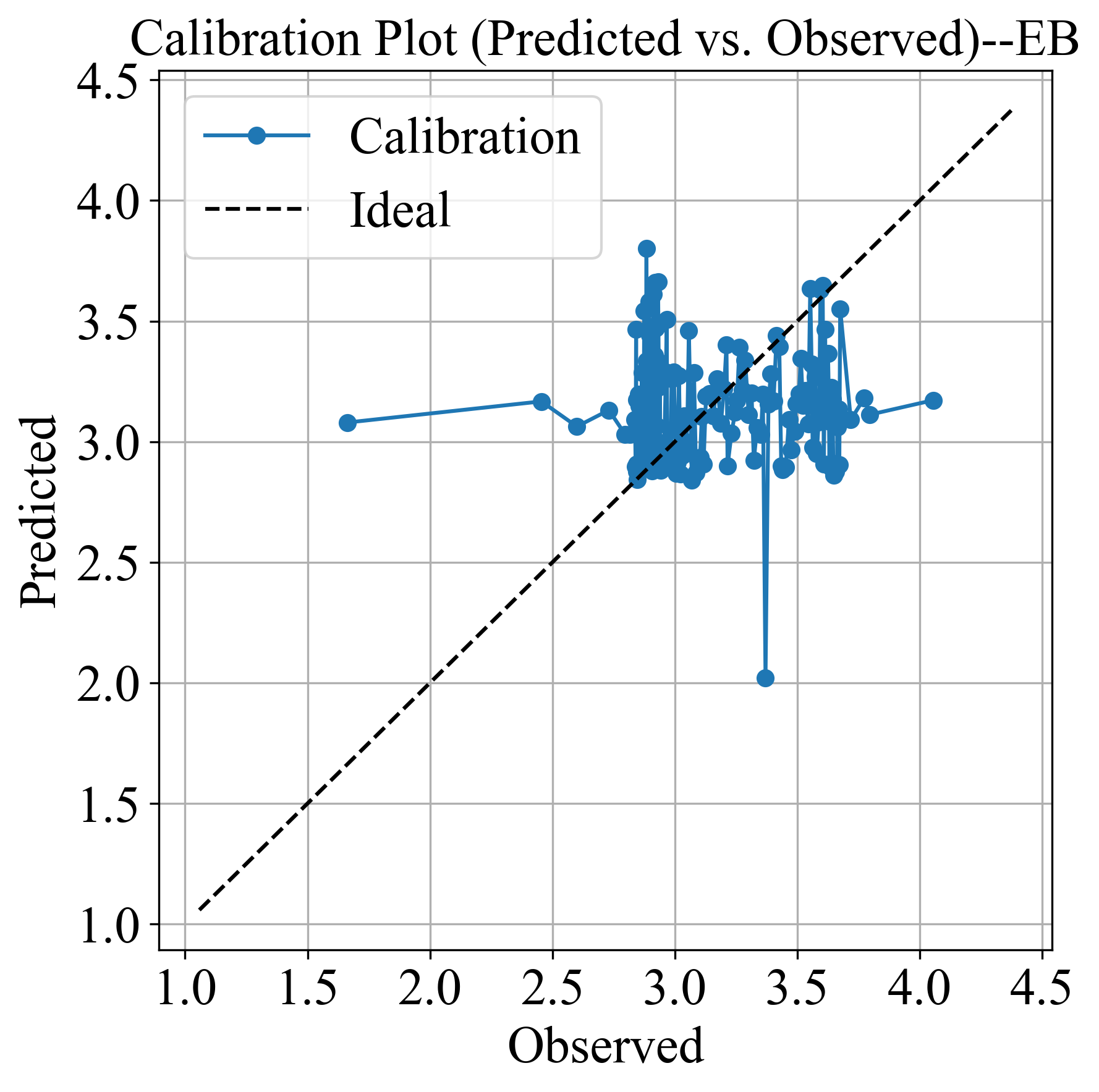EB | 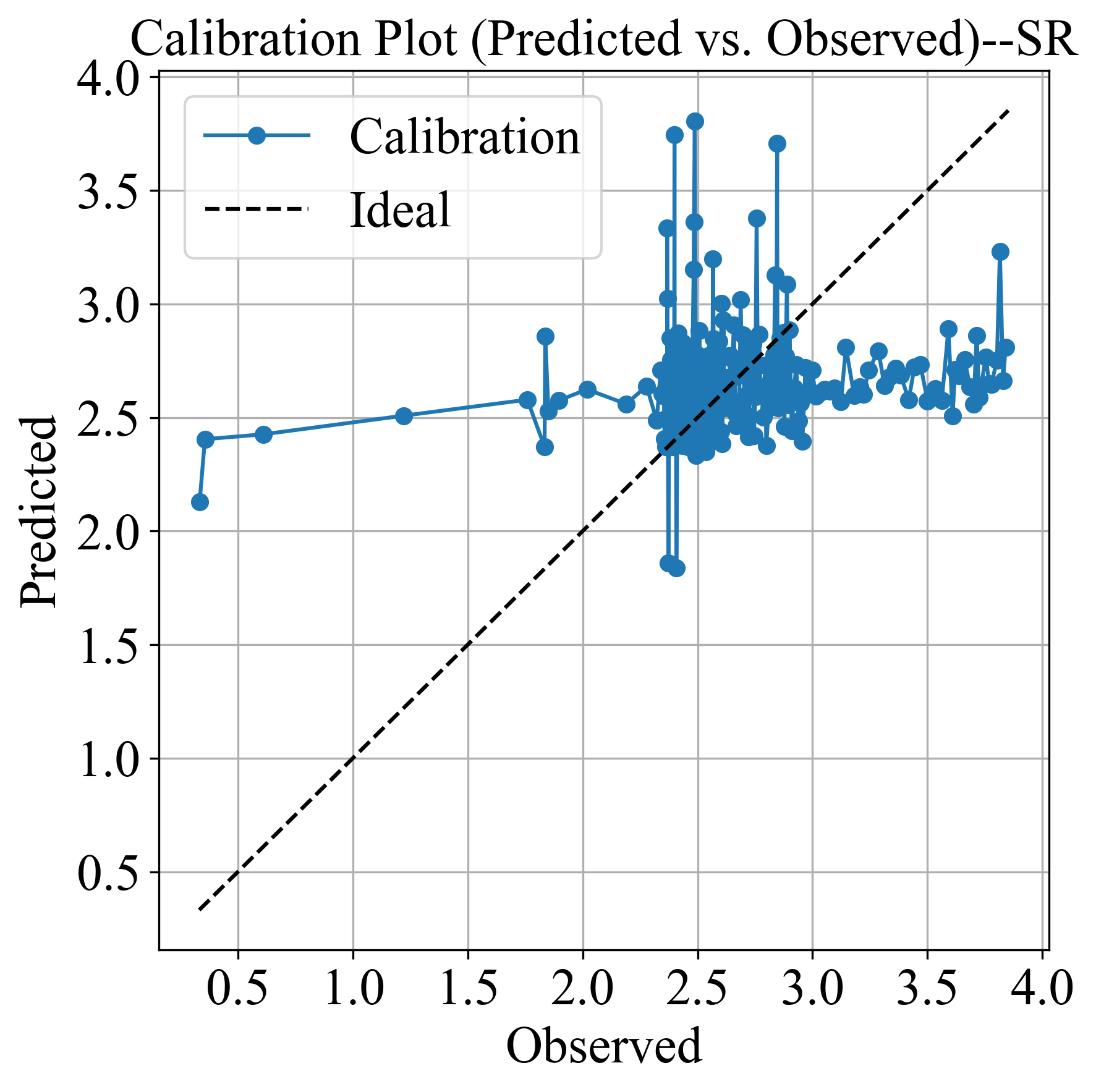SR |
| 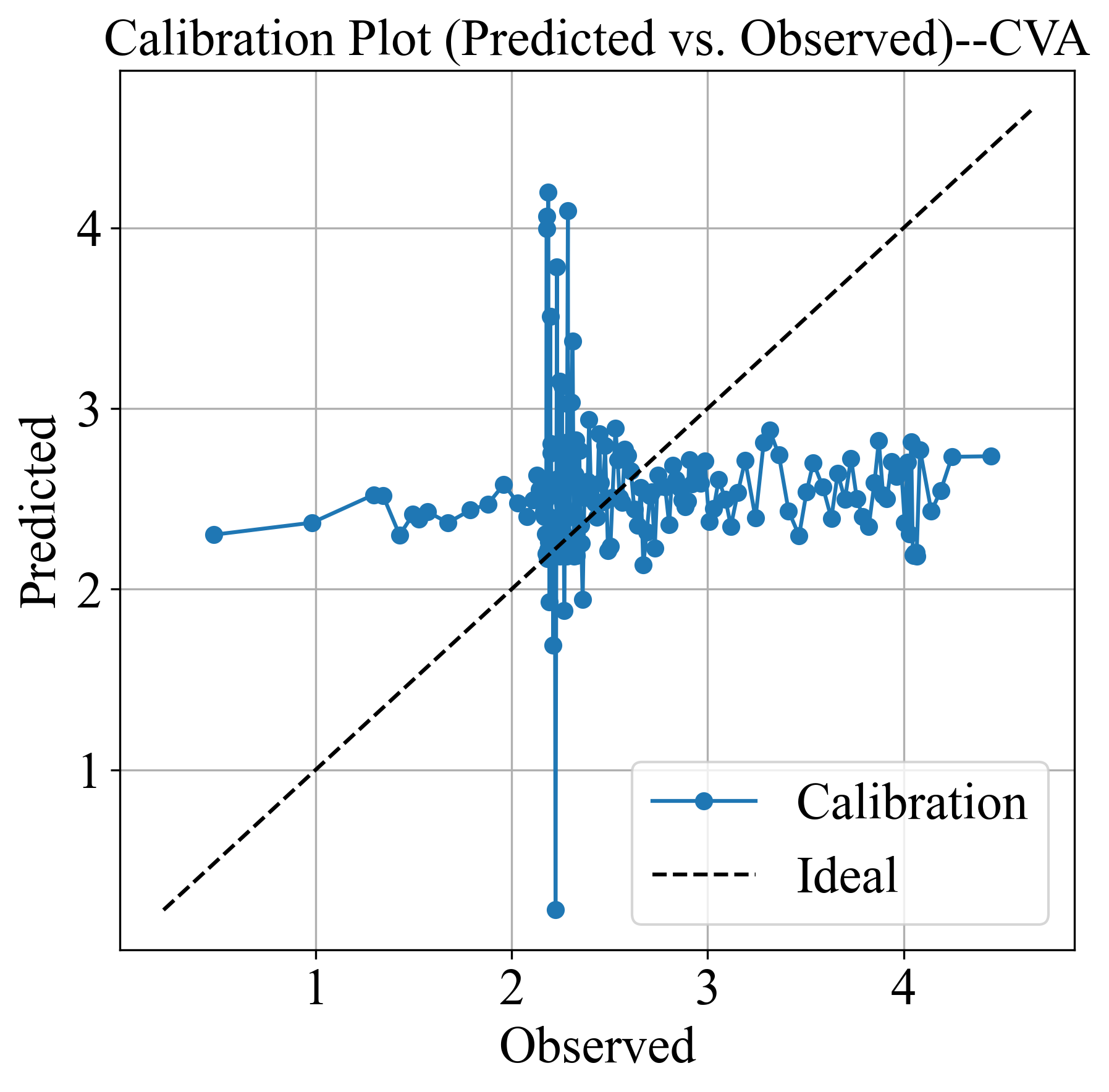CVA | 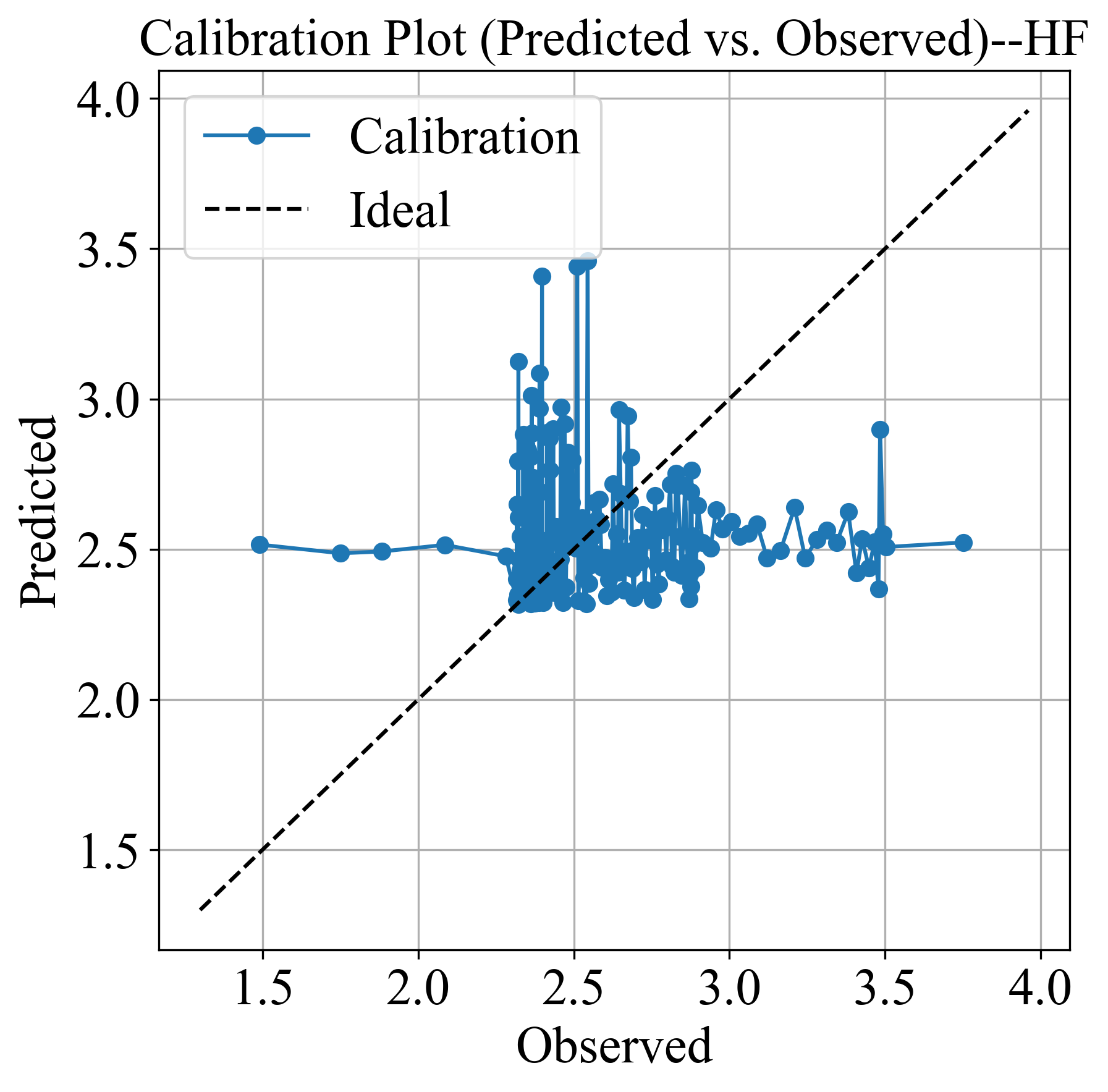HF |

Figure S2.1. Calibration plots of WT-LSTM predictions using 24-hour vital sign inputs

## S3. TRIPOD+ AI Checklist for Transparent Model Reporting

To ensure transparency and reproducibility, we provide a completed checklist based on the TRIPOD+ AI reporting guidelines [2]. This checklist documents how key elements of model development, evaluation, and reporting were addressed in our study. It serves as a reference for both future studies building on this work and for assessing adherence to emerging best practices in medical AI modeling.

Table S3.1. TRIPOD + AI Checklist for Transparent Model Reporting

| **Item** | **How It Was Addressed in This Study** |
| --- | --- |
| **Class imbalance** | As this study predicted continuous length of stay (LOS) rather than a binary classification, traditional class imbalance was not a primary issue. However, the right-skewed distribution of LOS was addressed by maintaining this distribution across training, validation, and test sets. |
| **Care pathway** | The study aligns with the ICU care pathway by focusing on early LOS prediction using vital signs available within the first hours of ICU admission, supporting resource allocation and discharge planning decisions. |
| **Discrimination** | Model discrimination was quantified using Mean Squared Error (MSE) for the continuous outcome (LOS). Performance was compared across multiple cohorts and baseline models. |
| **Evaluation or test data** | A held-out test set, stratified and sampled from the same population as training data, was used exclusively for model evaluation. There was no overlap of participants across training, validation, and test sets. |
| **Fairness** | This study did not stratify performance by sensitive attributes (e.g., race, sex, socioeconomic status). Fairness remains a consideration for future work with enriched datasets. |
| **Hyperparameters** | Key hyperparameters included learning rate (tuned across [0.08–0.15]), hidden units (15), and number of LSTM layers (1). |
| **Hyperparameter tuning** | Grid search was applied across multiple random seeds and learning rates (0.08, 0.1, 0.12, 0.15) using validation loss to select the best-performing model configuration. |
| **Internal validation** | Internal validation was conducted using 30 train-validation-test splits with different random seeds to assess robustness and stability of performance. |
| **Machine learning** | This study applied supervised deep learning, specifically a Wavelet-Transformed Long Short-Term Memory (WT-LSTM) model, to predict ICU length of stay based on multivariate time series vital sign data. |
| **Model evaluation** | Model performance was evaluated using MSE across ten patient cohorts and multiple time windows (3h, 6h, 12h, 24h). Comparative evaluations with LSTM, BiLSTM, and APACHE IV were conducted. |
| **Outcome** | The target outcome was length of stay in the ICU, expressed in days, and modeled as a continuous variable. |
| **Predictor** | Predictors included real-time physiological signals: heart rate, respiratory rate, and oxygen saturation (SaO₂). No demographic or lab data were used. |
| **Training or development data** | Model training was conducted on data from the top 10 most common ICU diagnoses in the eICU database, with vital signs as inputs. The total dataset was split into training (56.25%), validation (18.75%), and test (25%) sets. |

References

1. Moreno RP, Metnitz PGH, Almeida E, Jordan B, Bauer P, Campos RA, Iapichino G, Edbrooke D, Capuzzo M, Le Gall J-R, on behalf of the SAPS 3 Investigators. SAPS 3—From evaluation of the patient to evaluation of the intensive care unit. Part 2: Development of a prognostic model for hospital mortality at ICU admission. Intensive Care Med 2005 Oct;31(10):1345–1355. doi: 10.1007/s00134-005-2763-5

2. Collins GS, Moons KGM, Dhiman P, Riley RD, Beam AL, Van Calster B, Ghassemi M, Liu X, Reitsma JB, Van Smeden M, Boulesteix A-L, Camaradou JC, Celi LA, Denaxas S, Denniston AK, Glocker B, Golub RM, Harvey H, Heinze G, Hoffman MM, Kengne AP, Lam E, Lee N, Loder EW, Maier-Hein L, Mateen BA, McCradden MD, Oakden-Rayner L, Ordish J, Parnell R, Rose S, Singh K, Wynants L, Logullo P. TRIPOD+AI statement: updated guidance for reporting clinical prediction models that use regression or machine learning methods. BMJ 2024 Apr 16;e078378. doi: 10.1136/bmj-2023-078378
